# The combined action of mast cell chymase, tryptase and carboxypeptidase A3 protects against melanoma colonization of the lung

**DOI:** 10.18632/oncotarget.15339

**Published:** 2017-02-15

**Authors:** Mirjana Grujic, Aida Paivandy, Ann-Marie Gustafson, Allan R. Thomsen, Helena Öhrvik, Gunnar Pejler

**Affiliations:** ^1^ Uppsala University, Department of Medical Biochemistry and Microbiology, Uppsala, Sweden; ^2^ University of Copenhagen, Department of Immunology and Microbiology, Copenhagen, Denmark; ^3^ Swedish University of Agricultural Sciences, Department of Anatomy, Physiology and Biochemistry, Uppsala, Sweden

**Keywords:** mast cells, chymase, tryptase, carboxypeptidase A3, CD1d

## Abstract

Mast cell secretory granules are densely packed with various bioactive mediators including proteases of chymase, tryptase and CPA3 type. Previous studies have indicated that mast cells can affect the outcome of melanoma but the contribution of the mast cell granule proteases to such effects has not been clear. Here we addressed this issue by assessing mice lacking either the chymase Mcpt4, the tryptase Mcpt6 or carboxypeptidase A3 (Cpa3), as well as mice simultaneously lacking all three proteases, in a model of melanoma dissemination from blood to the lung. Although mice with individual deficiency in the respective proteases did not differ significantly from wildtype mice in the extent of melanoma colonization, mice with multiple protease deficiency (Mcpt4/Mcpt6/Cpa3-deficient) exhibited a higher extent of melanoma colonization in lungs as compared to wildtype animals. This was supported by higher expression of melanoma-specific genes in lungs of Mcpt4/Mcpt6/CPA3-deficient vs. wildtype mice. Cytokine profiling showed that the levels of CXCL16, a chemokine with effects on T cell populations and NKT cells, were significantly lower in lungs of Mcpt4/Mcpt6/Cpa3-deficient animals vs. controls, suggesting that multiple mast cell protease deficiency might affect T cell or NKT cell populations. In line with this, we found that the Mcpt4/Mcpt6/Cpa3-deficiency was associated with a reduction in cells expressing CD1d, a MHC class 1-like molecule that is crucial for presenting antigen to invariant NKT (iNKT) cells. Together, these findings indicate a protective role of mast cell-specific proteases in melanoma dissemination, and suggest that this effect involves a CXCL16/CD1d/NKT cell axis.

## INTRODUCTION

Mast cells (MCs) are hematopoietic cells that are present as resident populations in virtually all tissues of the body, with preponderance at sites close to the exterior such as skin and mucosal surfaces of the lung and gut [1]. MCs are characterized by a large content of secretory granules, which are densely packed with a variety of preformed compounds, including histamine, serotonin, cytokines, lysosomal enzymes, growth factors, serglycin proteoglycans and various MC-restricted proteases [2]. The MC-restricted proteases encompass tryptases, chymases and carboxypeptidase A3 (CPA3), of which the former two are serine proteases whereas CPA3 is a Zn-containing metalloprotease [3–5]. These proteases are all stored in enzymatically active form and when MCs are activated to degranulate, e.g. by IgE receptor crosslinking [6] or by engagement of the MRGPRX2/MRGPRB2 receptor [7], large amounts of these proteases are released to the cell exterior and can exert massive proteolytic action in the tissue [3, 4].

MCs have been implicated in a variety of pathological settings, most notably allergic conditions, but also in a range of additional pathologies such as arthritis, atherosclerosis, kidney inflammation and bacterial infection [8]. There is also substantial documentation, mainly from clinical studies, suggesting that MCs can have a role in malignant disorders, including squamous carcinomas, nodular sclerotic-type Hodgkin’s lymphoma, Waldenströms macroglobulinemia, breast cancer, prostate cancer and melanoma (reviewed in [9–13]).

In the case of melanoma, there is substantial documentation from both clinical studies and from animal models supporting that MCs participate in this pathology [14–22] (reviewed in [12]). However, there is still only little knowledge of the mechanism(s) by which MCs contribute to this malignancy. One important issue that has not been resolved is if the MC-restricted proteases contribute to melanoma pathogenesis. Clearly, since the MC proteases are known to cleave a variety of compounds of potential importance for tumor growth/dissemination [3, 4], there is a potential that melanoma pathology could be influenced by their release from MCs. Here we addressed this possibility by evaluating mice deficient in chymase (Mcpt4^−/−^), tryptase (Mcpt6^−/−^) or CPA3 (Cpa3^−/−^) in a model of melanoma colonization of the lung. Considering the possibility that the MC proteases may act in concert in the degradation of their substrates, we also evaluated mice with combined deficiency in Mcpt4, Mcpt6 and Cpa3. We show that melanoma colonization of the lungs is enhanced in animals with simultaneous absence of chymase, tryptase and CPA3, suggesting a protective role of the MC-restricted proteases. Moreover, our data suggest that the enhanced melanoma colonization seen in Mcpt4/Mcpt6/Cpa3-deficient animals is associated with a defective CXCL16/CD1d/iNKT cell axis.

## RESULTS

### The individual absence of chymase, tryptase or CPA3 does not affect melanoma colonization of the lung

To address the role of MC chymase in the melanoma model we assessed mice deficient in Mcpt4 (also known as mouse mast cell protease 4 (mMCP4)). Mcpt4 is the murine functional homologue to human chymase (CMA1) and is the dominating enzyme with chymotrypsin-like activity in various tissues including skin and lung [23, 24]. We also assessed animals lacking Mcpt6 (also known as mMCP6), a tetrameric tryptase that is similar in structure and function to human β-tryptase [25]. In addition we assessed animals deficient in CPA3 [26].

**Figure 1:**
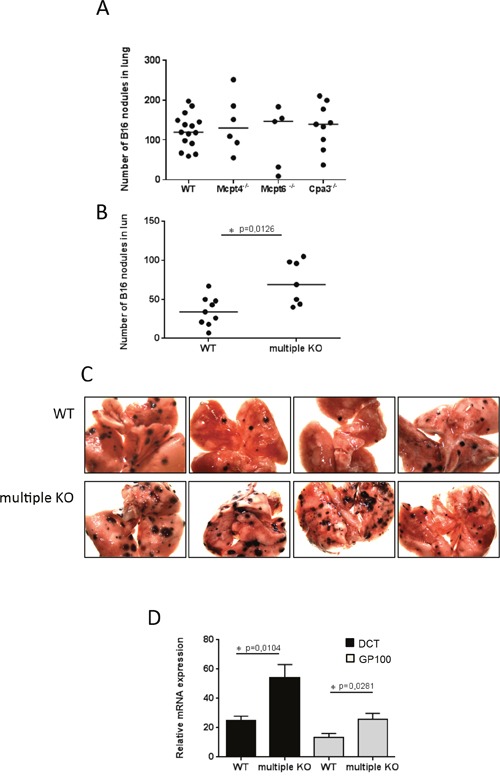
Multiple KO mice have higher tumor load in lungs compared to WT mice 0.25 million of B16F10 cells were injected i.v. into WT, single KO or into multiprotease-deficient (multiple KO) mice. On day 14 post injection, the numbers of tumor nodules were quantified **A, B**. Results are presented as medians (n = 5-15). **C**. Representative images of lungs from WT and multiple KO mice. Note the higher numbers of tumor nodules in multiple KO compared to WT lungs. **D**. Total RNA was isolated from lungs on day14 post injection and subjected to qPCR analysis of the expression of melanoma-specific genes (DCT, black bars; GP100, gray bars). Expression of genes was evaluated relative to GAPDH and normalized to naïve mice. Results are presented as mean ± SEM; n = 8. Statistical calculations were performed using the Mann Whitney test.

To address the role of these MC proteases in melanoma we used a model in which B16F10 melanoma cells are injected intravenously (i.v.), followed by an assessment of melanoma colonization of the lung. Fourteen days after i.v. administration of the melanoma cells, tumor nodules were enumerated in the lungs. However, there was no significant difference in the extent of melanoma colonization of lungs when comparing wildtype (WT), Mcpt4^−/−^, Mcpt6^−/−^ and Cpa3^−/−^ mice (Figure 1A). This suggests that the individual absence of Mcpt4, Mcpt6 or CPA3, respectively, does not affect the outcome in this model of melanoma colonization.

### Increased melanoma colonization of lungs in mice with simultaneous absence of chymase, tryptase and CPA3

Although the individual absence of Mcpt4, Mcpt6 or CPA3 did not affect melanoma dissemination to the lung, we considered the possibility that the combined action of these proteases can have an impact on melanoma colonization. This would be in agreement with previous studies showing that different MC proteases can act in concert in the degradation of selected proteins [4, 27, 28]. To evaluate this possibility we made use of mice with combined deficiency in Mcpt4, Mcpt6 and CPA3 [29]. Notably, the absence of CPA3 is known to cause a posttranslational defect in the storage of Mcpt5 (also known as mMCP5) [26], a MC-restricted protease that is structurally similar to CMA1 but has elastase-like cleavage specificity [30]. Thus, at the protein level, the Mcpt4/Mcpt6/Cpa3-deficient strain also lacks Mcpt5.

When assessing the Mcpt4/Mcpt6/Cpa3 multiple knockout (KO) strain in our model, we noted that the melanoma colonization of lung tissue was markedly higher than in WT mice (Figure 1B-1C), suggesting that the combined action of Mcpt4, Mcpt6, Cpa3 and Mcpt5 is protective against melanoma dissemination to the lung. To substantiate this finding we also used quantitative real time PCR (qPCR) to measure the expression of two melanoma-specific genes, Dct and Gp100, in lungs of WT vs. multiple KO mice. As seen in Figure 1D, the increased melanoma colonization of lungs from multiple KO animals was accompanied by significantly higher levels of mRNA representing Dct and Gp100. Hence, these findings reinforce that the combined action of Mcpt4, Mcpt5, Mcpt6 and Cpa3 protects against melanoma colonization of lungs.

**Figure 2:**
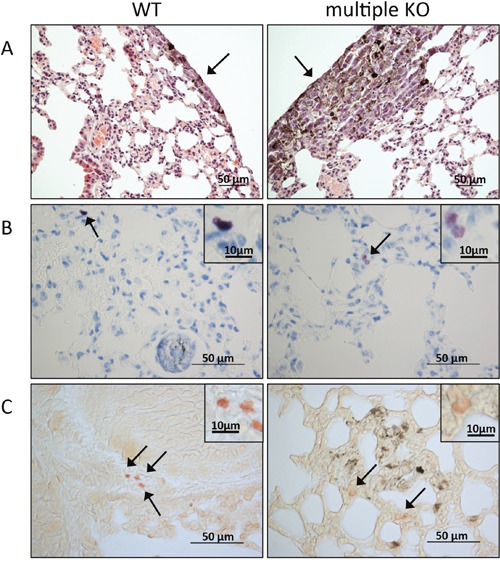
Histological analysis of lung tissue On day 14 post B16F10 cell injection, lungs were paraffin imbedded, sectioned and stained with hematoxylin and eosin **A**. toluidine blue **B**. or for chloroacetate esterase activity **C**. (A) Arrows point to tumor areas. Note a larger tumor area in the section from multiple KO mice (right) in comparison with WT controls (left); original magnification x 200; bar = 50 μm. (B) Toluidine blue-positive MCs (marked by arrows) are present in lung sections from both WT (left) and multiple KO (right). Inserts show a higher magnification of the toluidine blue-stained MCs; note that MCs in lungs of multiple KO mice stain less intensely in comparison with WT MCs. (C) Chloroacetate esterase staining shows the presence of chymase-positive MCs in lung of both WT (left) and multiple KO (right) mice (marked by arrows): insets show larger magnifications of chloroacetate esterase-stained MCs; note that MCs in lungs from multiple KO mice stain less intensely with chloroacetate esterase, indicating lower content of chymase activity. Original magnification x 400; bars = 50 μm; bars in the inserts =10 μm.

A histological examination of lungs from WT vs. multiple KO animals confirmed extensive establishment of melanoma cells in lungs of animals that had received i.v. administration of melanoma cells (Figure 2A). Toluidine blue staining revealed that MCs were present in lungs of both WT and multiple KO animals (Figure 2B). Notably, MCs in WT mice showed strong metachromatic staining, whereas MCs of multiple KO mice stained considerably weaker (Figure 2B). The latter is in agreement with the effect of Mcpt4/Mcpt6/Cpa3-deficiency on staining properties of MCs derived from the peritoneum and skin [29], and suggests that the simultaneous absence of Mcpt4/Mcpt6/Cpa3 affects the ability of lung MCs to store proteoglycans (see [29]). MCs were seen either at a distance from the melanoma areas or in the vicinity of the tumors (Figure 2B). We also stained the tissue sections with chloroacetate esterase, a reagent that stains for chymotrypsin-like enzymes such as chymases. As seen in Figure 2C, the chloroacetate esterase staining was strong in WT tissue but substantially diminished in lungs of multiple KO mice. These findings suggest that Mcpt4, i.e. the chymase that is depleted in the multiple KO animals, accounts for the major part of the chymotrypsin-like activity in lungs of melanoma-administered WT animals. The residual chloroacetate esterase activity seen in lungs of multiple KO animals is most likely reflecting chymase activity attributed to Mcpt1, a chymase known to be expressed by lung MCs [31].

### Absence of Mcpt4/Mcpt6/Cpa3 does not affect the early stages of melanoma colonization of lungs

The data above suggest that the simultaneous absence of Mcpt4/Mcpt6/Cpa3 results in increased melanoma colonization as manifested by the appearance of tumor nodules in lungs taken two weeks after melanoma cell administration. Next, we asked whether the absence of Mcpt4/Mcpt6/Cpa3 affects melanoma colonization at the early stages, i.e. before the appearance of visible tumor nodules. To address this we labeled melanoma cells with cell tracker orange CMRA and administered the labeled cells i.v.. After 9 h, single cell suspensions were prepared from lungs and were assessed by flow cytometry analysis for presence of CMRA-labeled (melanoma) cells. However, there was no significant effect of the combined absence of Mcpt4/Mcpt6/Cpa3 on the numbers of lung-associated melanoma cells at this stage of the malignant process (Supplementary Figure 1). Hence, the MC proteases do not impact on the early stages of melanoma colonization of the lungs.

### Expression of tumor-promoting genes in lungs of WT and Mcpt4/Mcpt6/Cpa3-deficient mice

**Figure 3:**
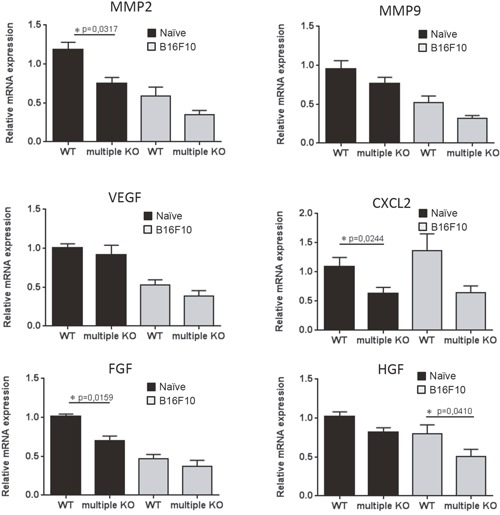
Expression of angiogenesis markers in lungs of naïve and melanoma-carrying mice Total RNA was isolated from lungs of naïve mice (black bars) and lungs of mice taken day 14 post i.v. injection of B16F10 melanoma cells (gray bars). Total RNA was subjected to qPCR analysis of angiogenesis markers. Expression of genes was evaluated relative to GAPDH and normalized to naïve mice. Results are presented as mean ± SEM; n = 5 (black bars), n = 7-8 (gray bars). Note that mice with tumors do not show increased expression of angiogenesis markers compared to naïve mice and that the groups with tumors show similar expression of the markers. Statistical calculations were performed using the Mann Whitney test.

In order to approach the mechanism by which the combined absence of Mcpt4/Mcpt6/Cpa3 could affect melanoma colonization, we first assessed the expression of various genes known to have an impact on malignant progression/dissemination. These analyses revealed that there was no difference in the expression of matrix metalloprotease 2 (MMP2), matrix metalloprotease 9 (MMP9), vascular endothelial growth factor (VEGF), CXCL2 or fibroblast growth factor (FGF) in melanoma-carrying WT vs. multiple KO animals (Figure 3). However, we noted a significant reduction in the expression of hepatocyte growth factor (HGF) in lungs from multiple KO vs. WT melanoma-carrying animals (Figure 3). Moreover, we noted a lower expression of MMP2, FGF and CXCL2 in naïve multiple KO animals vs. WT controls (Figure 3).

### Lower levels of CXCL16 in lungs of Mcpt4/Mcpt6/Cpa3-deficient mice

**Figure 4:**
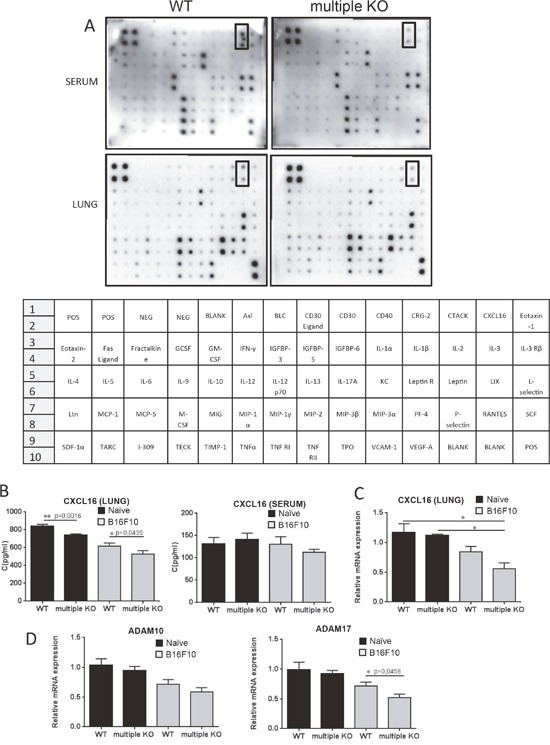
Lower levels of CXCL16 in lungs of multiple KO mice **A**. B16F10 melanoma cells were injected i.v. into either WT or multiple KO mice. On day14 post injection, mice were sacrificed, blood and lungs were collected (n = 3-4/genotype), and protein array (detecting 62 mouse proteins; setup indicated below the panel) analysis was performed on serum samples and lung homogenates as indicated. Squares represent CXCL16; note the lower amounts of CXCL16 in samples from multiple KO mice. **B**. Mouse CXCL16 ELISA was performed on lung homogenates (n = 9-10/genotype) and serum (n = 4-6/genotype) from naïve mice (black bars) or mice injected with B16F10 melanoma cells (gray bars). Results are presented as mean ± SEM. **C**. qPCR analysis of CXCL16 gene expression was performed on lung tissue from naïve (black bars, n = 4) and B16F10 melanoma-injected mice (gray bars, n = 8). Expression of genes was evaluated relative to GAPDH and normalized to naïve mice. Results are presented as mean ± SEM (Kruskal-Wallis test). **D**. qPCR analysis of ADAM10 and ADAM17 expression was performed on lung tissue from naïve (black bars; n = 4-5) and B16F10 melanoma-injected mice (gray bars; n = 8). Expression of genes was evaluated relative to GAPDH and normalized to naïve mice. Results are presented as mean ± SEM. Statistical calculations were performed using the Mann Whitney test.

As a next approach for dissecting the mechanism underlying the protective role of Mcpt4/Mcpt6/Cpa3 in melanoma colonization, we assessed the levels of various cytokines and chemokines in serum of WT vs. multiple KO mice using an antibody-based filter array. As seen in Figure 4A, the majority of the compounds included in the array did not differ between serum from WT vs. multiple KO mice. However, the filter array analysis indicated that the levels of CXCL16, a chemokine with known ability to attract T cell populations and NKT cells [32], was reduced in serum of multiple KO animals. Similarly, the filter array approach indicated diminished levels of CXCL16 in lung tissue of multiple KO vs. WT animals (Figure 4A). To verify these findings with a quantitative method we used ELISA. Indeed, ELISA measurements confirmed that the levels of CXCL16 were significantly lower in lungs of multiple KO vs. WT animals, whereas we were not able to confirm the effect of combined Mcpt4/Mcpt6/Cpa3-deficiency on the levels of CXCL16 in serum (Figure 4B). In line with these findings, qPCR analysis revealed that the expression of the CXCL16 gene was significantly lower in lungs of multiple KO animals that had received melanoma cells, as compared with naïve WT or Mcpt4/Mcpt6/Cpa3-deficient animals (Figure 4C). In contrast, there was no significant difference in CXCL16 gene expression when comparing melanoma-carrying WT animals vs. naïve WT or Mcpt4/Mcpt6/Cpa3-deficient animals (Figure 4C).

CXCL16 is unique (together with CXCL1) among chemokines by being expressed as a transmembrane protein [32]. Previous studies have shown that CXCL16 can be shed from the cell surface to generate a soluble chemokine, and it has been shown that this is mediated by classical sheddases such as a disintegrin and metalloproteinase 10 (ADAM10) or ADAM17 [32–34]. To evaluate the possibility that the differential levels of CXCL16 in WT vs. multiple KO animals is attributed to effects on the expression of sheddases, we compared the expression of the ADAM10 and ADAM17 genes in lungs of WT vs. multiple KO animals. These analyses revealed a slight but significant decrease in the expression of ADAM17 in lungs from multiple KO vs. WT animals, whereas the expression of ADAM10 was not affected by the absence of Mcpt4/Mcpt6/Cpa3 (Figure 4D).

### Absence of Mcpt4/Mcpt6/Cpa3 is associated with a decrease in CD1d-expressing cells

**Figure 5:**
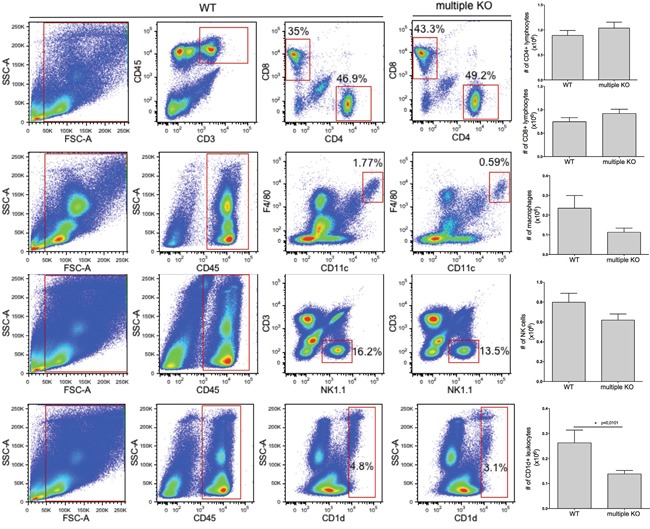
Decreased numbers of CD1d-expressing lung cells in multiple KO mice Representative dot plots and numbers of **A**. CD45^+^CD3^+^CD4^+^/CD8a^+^ lymphocytes, **B**. CD45^+^CD11c^+^F4/80^+^ (macrophages), **C**. CD45^+^CD3^−^NK1.1^+^ (NK cells) and **D**. CD45^+^CD1d^+^ cells in lungs of melanoma-carrying WT or multiple KO mice (day 14 post melanoma administration). The bars show the mean ± SEM of 10-11 individual mice per group pooled from two independent experiments. Mann-Whitney test; * p < 0.05.

**Figure 6:**
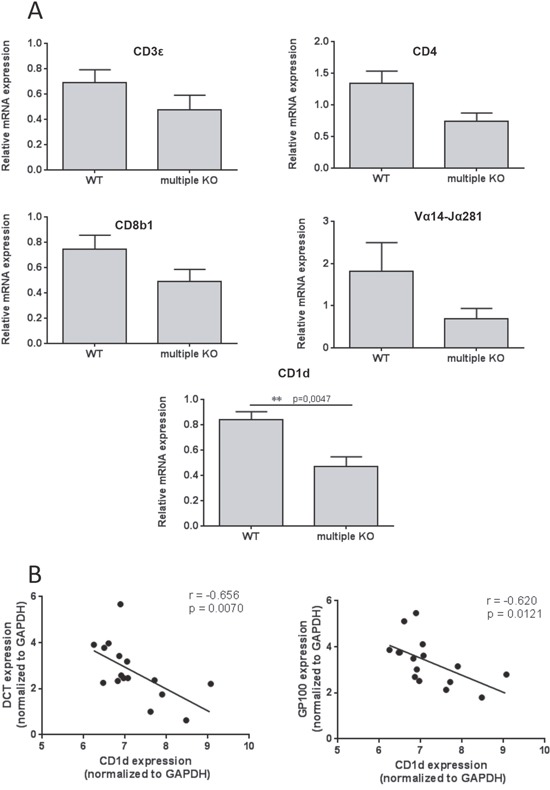
Decreased expression of CD1d in multiple KO mice **A**. qPCR analysis of CD3ε, CD4, CD8b1, Vα14-Jα281 and CD1d expression was performed on lung tissues from B16F10 melanoma-carrying WT or multiple KO mice (n = 6-8). Expression of genes was evaluated relative to GAPDH and normalized to naïve mice. Results are presented as mean ± SEM. Statistical calculations were performed using the Mann Whitney test. **B**. Correlation between the expression of CD1d with expression of melanoma-specific genes: DCT and GP100; Nonparametric Spearman correlation.

Next we considered the possibility that the altered melanoma colonization in Mcpt4/Mcpt6/Cpa3-deficient animals might be reflected by effects on leukocyte recruitment into the lungs. To assess this we generated single cell suspensions of lungs from melanoma-administered WT and multiple KO animals, and used flow cytometry analysis to quantify various CD45^+^ leukocyte populations. The absence of Mcpt4/Mcpt6/Cpa3 did not affect the percentage of CD3^+^CD4^+^, CD3^+^CD8^+^, F4/80^+^Cd11c^+^ (macrophages) or CD3^−^NK1.1^+^ (NK) cells (Figure 5). However, we noted a significant reduction of the CD1d^+^ population in lungs of multiple KO mice vs. WT controls (Figure 5). In agreement with this finding, qPCR analysis revealed a significant reduction in the expression of the CD1d gene in multiple KO vs. WT melanoma-carrying mice (Figure 6A). In contrast, the expression of the CD3, CD4, CD8 or Vα14-Jα281 (component of the T cell receptor expressed by iNKT cells) genes did not differ in lungs recovered from WT vs. multiple KO animals (Figure 6A). Hence, the high melanoma burden of Mcpt4/Mcpt6/Cpa3-deficient animals is accompanied by low expression of CD1d, introducing the possibility that CD1d might have a role in regulating melanoma colonization of the lung. In agreement with this notion, the expression of the CD1d gene correlated negatively with the expression of melanoma-specific genes (Figure 6B).

## DISCUSSION

It has been recognized for a long time that MC presence is a common clinical feature of malignant melanoma (see Introduction). In a previous study on this topic, the presence of MCs was correlated with poor prognosis [15], suggesting a detrimental role of MCs in this type of malignancy. A detrimental role of MCs in melanoma is also supported by experimental studies performed on mice lacking MCs altogether. The latter studies have been based on animals where the absence of MCs is caused either by mutations in c-kit [19, 20] or by MC-specific expression of Cre recombinase leading to diphteria toxin-mediated cellular suicide [18]. However, although these previous efforts support a role of MCs in melanoma, the mechanisms by which MCs impact on this type of pathology are not fully understood. One favored scenario though is that MCs might contribute by expressing pro-angiogenic factors such as VEGF [16], bFGF [35] and histamine [36], whereas other studies have suggested that MCs may account for TIM-3-mediated immunosuppression [37].

In contrast to the prevailing notion that MCs as such promote melanoma, we here report that the combined action of MC-restricted granule-localized proteases confers protection against melanoma colonization. Notably, our present findings are in agreement with a recent clinical study where it was shown that the presence of MC chymase and tryptase correlated with good prognosis [21]. Hence, the present findings together with previous observations suggest that MCs can have both tumor-promoting and -protective activities. As an explanation for this apparent dichotomy, we can envision that the tumor-suppressing activities of the MC proteases can balance tumor-promoting activities of other compounds expressed by MCs. For example, one potential scenario is that MCs express tumor-promoting chemokines/growth factors, and that the MC proteases can regulate the levels of such compounds by proteolytic action. The absence of the MC protease system will accordingly lead to an elevation of factors with tumor-promoting activity; however, the identities of the *in vivo* substrates for Mcpt4/Mcpt6/Cpa3 during the course of melanoma progression remain to be revealed. The suggested scenario is thus in analogy with previous findings demonstrating that MC chymase can have an anti-inflammatory role in allergic airway inflammation whereas MCs as such promote this condition [24, 38, 39]. Another example is the reported detrimental function of MC of chymase in immune complex-mediated glomerulonephritis, whereas MCs overall promote the pathology in this setting [40].

The proteases assessed in this study, i.e. Mcpt4, Mcpt6 and CPA3 (and indirectly Mcpt5), are all expressed by MCs of the connective tissue subtype (CTMCs), and our findings thus indicate the proteases expressed by this subclass of MCs influence melanoma colonization. However, lungs are also populated by mucosal type MCs (MMCs), which express Mcpt1 and Mcpt2. Adding complexity to this issue, it has been reported that mouse lung MCs represent a mixed phenotype, expressing both classical CTMC proteases but also Mcpt1 and Mcpt2 [31]. At present we do not know whether the MMC proteases, i.e., Mcpt1 or Mcpt2, influence melanoma. However, an assessment of Mcpt1 and Mcpt2 KO animals in melanoma colonization would be clearly warranted.

As regards the molecular mechanism by which the mast cell proteases affect melanoma dissemination, our findings reveal that the absence of Mcpt4/Mcpt6/Cpa3 is associated with a decrease in ADAM17 expression. ADAM17 is a well-known sheddase having the ability to cleave off the ectodomains of a numerous cell surface proteins, including CXCL16 [32–34]. It is therefore plausible that reduced ADAM17 activity in the multiple KO animals could lead to less efficient shedding of CXCL16, thereby explaining the observed reduction in CXCL16 levels (Figure 4). We also noted that the absence of Mcpt4/Mcpt6/CPA3 was associated with alterations in the expression a range of other genes of potential impact on the melanoma colonization processes: HGF, MMP2, FGF, CXCL2. Possibly, the altered expression of these genes could thus contribute to the enhanced susceptibility of multiple KO animals to lung melanoma colonization.

Interestingly, it was shown in a previous study, utilizing a subcutaneous model of melanoma, that mice with defective expression of NDST2 developed larger metastatic lesions than did WT mice [41]. NDST2 is an essential enzyme in the biosynthesis of heparin in MCs [42, 43] and the authors suggested that the phenotype of the NDST2 KO was linked to the anticoagulant activity of heparin [41]. In our initial characterization of the multiple KO mice, we noted that the combined absence of Mcpt4/Mcpt6/CPA3 led to a reduction in the content of heparin in MCs [29], i.e. similar to mast cells lacking NDST2. Hence, it is possible that the reduction of heparin content in MCs derived from the multiple KO mice might contribute to the enhanced susceptibility to melanoma. However, the reduction of heparin in MCs from multiple KO mice is only partial (∼50%) and we therefore favor the notion that it is the actual deficiency in proteases, rather than the reduction in heparin content, that is the major cause for the increased susceptibility of the multiple KO mice to melanoma. As an alternative scenario explaining for the effect of NDST2 on melanoma, it is noteworthy that heparin is crucial for promoting the storage of MC proteases, including Mcpt4, Mcpt5, Mcpt6 and Cpa3 [42]. Hence, a plausible explanation for the findings presented in [41] is that the increased melanoma burden seen in NDST2 KO animals is due to their deficiency in the respective NDST2-dependent MC proteases (i.e. Mcpt4, Mcpt5, Mcpt6, CPA3). Clearly, the latter scenario is in agreement with the findings presented in this study.

Our findings indicate that the levels of CXCL16 are significantly reduced in multiple KO animals. CXCL16 has previously been shown to impact on T cells, plasma cells and iNKT cells [32]. Hence, a plausible scenario is that the impaired ability of multiple KO mice to combat melanoma is due to effects on cell populations that are dependent on CXCL16. In line with this, we found that the combined deficiency of Mcpt4/Mcpt6/Cpa3 was associated with a reduction in the levels of CD1d. CD1d is expressed by a multitude of cell types but is specific for presentation of glycolipid antigens to iNKT cells [44, 45], a cell population previously shown to be an important player in the host’s anti-tumor defense [46]. Our findings thus suggest that the iNKT cell population may be impaired in mice lacking Mcpt4/Mcpt6/Cpa3, which could lead to impaired protection against melanoma dissemination. This notion was supported by our observed negative correlation between CD1d expression and melanoma burden.

Interestingly, single defects in any of the MC proteases were insufficient to cause increased melanoma progression whereas the simultaneous depletion of Mcpt4/Mcpt6/Cpa3 at the gene level (combined with post-translational defects in Mcpt5 storage) led to a significant increase in melanoma burden. One explanation for this might be that the individual MC proteases have distinct proteolytic targets during melanoma progression, but that the degradation of those targets alone is not sufficient to provide protection. Accordingly, the simultaneous absence of the various MC proteases could lead to the accumulation of several compounds whose combined action may enhance melanoma progression. An alternative scenario is that the various MC proteases act in concert on the same proteolytic targets, in this way resulting in a more rapid and extensive degradation of the respective substrate(s) than would be achieved by the individual MC proteases alone (see [4] for a discussion). Possibly, efficient degradation of target proteins, achieved by this mechanism, may be required to significantly affect melanoma progression.

## MATERIALS AND METHODS

### Mice

Wild type (WT), Mcpt4^−/−^, Mcpt6^−/−^, Cpa3^−/−^ and Mcpt4/Mcpt6/Cpa3-deficient mice were all on C57BL/6J genetic background. The Mcpt4^−/−^, Mcpt6^−/−^, Cpa3^−/−^ mice as well as the Mcpt4/Mcpt6/Cpa3-deficient mice show normal reproductive behavior and are fully viable. Eight- to 16-wk-old mice were used in all experiments. All experiments were approved by the Local Ethics Committee (Uppsala djurförsöksetiska nämnd; C 84/14).

### Tumor inoculation

The B16F10 melanoma cell line was obtained from ATCC (CRL-6475). Tumor cells were propagated in DMEM supplemented with 10% FBS, 1% L-glutamine and 1% penicillin/streptomycin solution. Prior to injection into the tail vein, cells reaching approximately 90% confluency were trypsinized, resuspended in Hanks’ balanced salt solution and counted using Trypan blue in order to adjust the cell concentration. 0.25 × 10^6^ B16F10 cells were injected into the tail vein. On day 14 post injection, mice were sacrificed and lung tissue to be used for flow cytometry analysis was flushed with PBS prior to harvesting. For histochemical analysis and evaluation of tumor nodules, the lung tissue was placed in 4% formalin (PBS buffered) solution. For quantitative real time RT-PCR (qPCR) analysis, protein array analysis or ELISA measurements, lungs were frozen on dry ice and stored at -80°C until use. Blood was collected both from B16F10-injected and naïve mice. Visible tumor nodules were enumerated and images were taken using a dissecting microscope (Leica EZ4 D; Leica Microsystems, Wetzlar, Germany).

When analyzing initial colonization of B16F10 cells in lungs, determined 9h post injection, melanoma cells were stained with cell tracker orange CMRA (Invitrogen, Carlsbad, CA) prior to the injection. For this, growing cells were first rinsed with serum-free medium and then incubated for 45 min with serum-free medium containing 10 μM CMRA, followed by trypsinization.

### RNA extraction and qPCR

Mice were euthanized; lungs were collected, frozen on dry ice and stored at -80°C until use. Whole lung tissue was homogenized in ∼1.7 ml of trizol reagent (Invitrogen) using polytron PT1200 (Kinematica AG, Luzern, Switzerland). The homogenates were centrifuged at 12,000g for 1 min and 500 μl of the supernatant (corresponding to ∼50 mg lung tissue) was used for total RNA isolation using Direct-zol RNA MiniPrep kit (The Epigenetics Company, Irvine, CA). Subsequently, total RNA concentration and purity was measured using a NanoDrop 1000 Spectrophotometer (Thermo Scientific, Wilmington, DE) and the ND-1000 V3.7.0 program. First-strand cDNA was synthesized using ∼100 ng RNA as template using the iScript cDNA synthesis kit (Bio-Rad, Hercules, CA), following the manufacturer’s instructions. Subsequently, qPCR was performed on a 7900 HT Fast Real-Time PCR System (Applied Biosystems, Foster City, CA) using 100 ng cDNA, 250-500 nM primers (see Table 1) and SYBR GreenER SuperMix (Invitrogen), following the PCR cycling conditions recommended by the manufacturer. Each sample was analyzed in duplicates and qPCR data analysis was performed using the SDS2.3 software. Gene expression levels were presented relative to the housekeeping gene Glyceraldehyde 3-phosphate dehydrogenase (GAPDH) and relative to naïve mouse.

**Table 1:** Primers used for qPCR analyses

Target	Forward primer (5`- 3`)	Reverse primer (5`- 3`)
GAPDH	CTC CCA CTC TTC CAC CTT CG	CCA CCA CCC TGT TGC TGT AG
DCT	TCC TCC ACT CTT TTA CAG ACG	ATT CGG TTG TGA CCA ATG GG
GP100	AGC ACC TGG AAC CAC ATC TA	GTT CCA GAG GGC TGT GTA GT
MMP2	GCG CTT TTC TCG AAT CCA T	GGG TAT CCA TCT CCA TGC TC
MMP9	CTG GAA CTC ACA CGA CAT CTT	TCC ACC TTG TTC ACC TCA TTT
VEGF	GGA GTC TGT GCT CTG GGA TT	AAC CAA CCT CCT CAA ACC GT
CXCL2	ACA TCC CAC CCA CAC AGT GAA	TCC TTC CAT GAA AGC CAT CCG
FGF	CCA CAC GTC AAA CTA CAA CTC C	TCG TTT CAG TGC CAC ATA CC
HGF	ATC CCA AAT CGT CCT GGT ATT T	CTG GCC TCT TCT ATG GCT ATT AC
CXCL16	CCT TGT CTC TTG CGT TCT TCC	TCC AAA GTA CCC TGC GGT ATC
ADAM10	AGC AAC ATC TGG GGA CAA	AAA GTT GGG CTT GGG ATC
ADAM17	GGT GGA CGG GAA AGA AGA AA	CTA TGT GGG CTA GAA CCC TAG A
CD1d	AAT CTG AAG CCC AGC AAA AGA A	TTA CTC CAA CGG TGA GTC TGC
CD3ε	AAC ACG TAC TTG TAC CTG AAA GCT C	GAT GAT TAT GGC TAC TGC TGT CA
CD4	AGG TGA TGG GAC CTA CCT CTC	GGG GCC ACC ACT TGA ACT AC
CD8b1	GAC GAA GCT GAC TGT GGT TGA	GCA GGC TGA GGG TGG TAA G
Tcra-V14	GAG AGA ACT GCG TCC TTC AAT	GAC TGT CAG GGA CAC AAG AC

### Histochemistry

Lung tissues fixed in 4% formalin (PBS buffered) were embedded in paraffin and sectioned into 6-μm sections. Samples were deparaffinized and stained either with hematoxylin and eosin, toluidine blue or chloroacetate esterase as previously described [47]. All images were generated using bright field using a Nikon 90i microscope (Nikon Instruments Europe, Amsterdam, Netherlands) and at an original magnification of 400x.

### Protein analysis, protein array and ELISA

For protein analysis, the Mouse Cytokine Antibody Array C3 (cat. # AAM-CYT-3-4, RayBiotech, Norcross, GA), detecting 62 mouse proteins, was used. Whole lungs from B16F10-injected mice were homogenized on ice with polytron PT1200 (Kinematica AG) in 1.9 ml of 1 x cell lysis buffer (cat. # AAM-CYT-3-4, RayBiotech) supplemented with complete protease inhibitor cocktail (Roche, Mannheim, Germany). Homogenates were centrifuged at 10,000g at 4°C for 5 min; the supernatants were aliquoted and stored at -80°C before use. Before protein array analysis, equal volumes (100 μl) of lung homogenates from 5 mice/genotype were mixed; the protein concentration of these mixed homogenates were determined using BCA1-KIT (Sigma, St. Louis, MO) and 500 μg of the samples were applied on the membrane. Serum samples were mixed in a similar manner and applied on the membrane. Membranes were exposed to HRS-Streptavidin and detection buffer, and scanned using a chemiluminiscence imaging system (BioRad CCD camera). Mouse CXCL16 ELISA (cat. # DY503, R&D Systems, Minneapolis, MN) was performed on lung homogenates and serum from naïve or B16F10-injected mice. Lung tissues were homogenized with polytron PT1200 in lysis buffer (100 mM Tris pH 8, 0.1 mM EDTA, 150 mM NaCl, 1% Nonidet-P40, 1mM NaH_2_PO_4_) with complete protease inhibitor cocktail (Roche) on ice. From each lung, samples corresponding to 500 μg protein were used for the analyses. Absorbance was determined using a microplate reader (Tecan Infinite 200; Tecan Austria, Grödig, Austria) and the Magellan V. 6.6 software.

### Flow cytometry

Lungs flushed with PBS were cut into small pieces, transferred into gentleMACS C tubes containing enzyme solution from the lung dissociation kit (Miltenyi Biotec, Bergisch Gladbach, Germany) and digested using gentleMACS Octo Dissociator with Heaters (Miltenyi Biotec). Next, the epithelial cells and cell debris were removed by resuspending cells in 10 ml of 44% Percoll (Sigma-Aldrich, St. Louis, MO) followed by centrifugation at 400 g, 4°C for 20 min using a Heraeus Megafuge 40R centrifuge (Thermo Fisher Scientific, Langenselbold, Germany). Lung cells were resuspended in 2% heat-inactivated FBS (Thermo Fisher Scientific, Waltham, MA) in PBS (FACS buffer), filtered through a 70 μm cell strainer (Corning, Corning, NY) and counted using Trypan blue. Two million cells/sample were incubated with mouse BD Fc Block (BD Biosciences, San Jose, CA) in FACS buffer, following the manufacturer’s recommendation. After 10 min incubation at 4°C, fluorochrome-conjugated antibodies in FACS buffer were added and the cells were stained for 20 min at 4°C in darkness. Cells were rinsed twice with FACS buffer and analyzed on a LSR Fortessa flow cytometer (BD Biosciences). The following antibodies were used: anti-CD45-APC (30-F11), CD3-FITC (17A2), CD1d-BV421 (1B1), CD4-PE (GK1.5), CD8a-PE-Cy7 (53-6.7), CD11c-BV421 (N418), NK1.1-BV711 (PK136) and F4/80-PE (T45-2342), all from BD Biosciences, (Franklin Lakes, NJ). Stained cells were analyzed using a LSR Fortessa flow cytometer (BD Biosciences), and data analysis was performed using the FlowJo software. FMO controls with appropriate isotype antibodies were used to set the gates.

### Statistical analysis

All analyses were performed in GraphPad Prism using the two-tailed unpaired Mann-Whitney test and Kruskal-Wallis test. Results shown are collected data from at least 2 independent experiments, presented as mean ± SEM. p values ≤0.05 were considered statistically significant.

## SUPPLEMENTARY FIGURE


