# Supplementary material for: The combined action of mast cell chymase, tryptase and carboxypeptidase A3 protects against melanoma colonization of the lung
Source: Oncotarget. 2017 Feb 15;8(15):25066–79. doi: 10.18632/oncotarget.15339 (PMC5421910; doi:10.18632/oncotarget.15339)
Supplement: Supplementary file 1 [file oncotarget-08-25066-s001.pdf]

The combined action of mast cell chymase, tryptase and carboxypeptidase A3 protects against melanoma colonization of the lung

SUPPLEMENTARY FIGURE

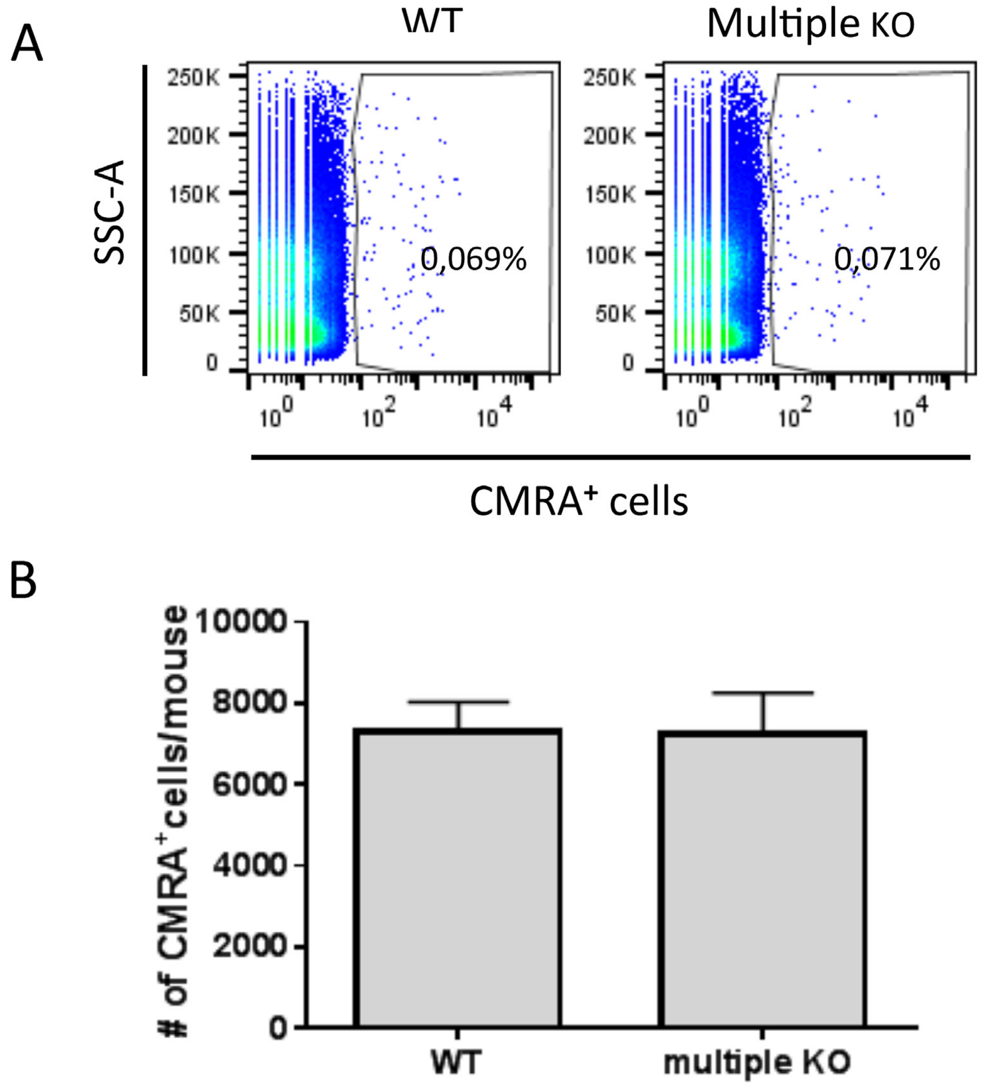

Supplementary Figure 1: Similar early colonization of tumor cells in lungs of WT and multiple KO mice. 0.25 million of B16F10 cells pre-stained with cell tracker orange CMRA were injected i.v. into WT or into multiple KO mice. Nine hours post injection, lungs were analyzed for the presence of CMRA$^+$ cells by flow cytometry. Representative dot plots **A.** and numbers **B.** of CMRA$^+$ cells, presented as mean ± SEM (n=4 /group).
